# Variability of orographic enhancement of precipitation in the Alpine region

**DOI:** 10.1038/s41598-019-49974-5

**Published:** 2019-09-16

**Authors:** Anna Napoli, Alice Crespi, Francesco Ragone, Maurizio Maugeri, Claudia Pasquero

**Affiliations:** 1 Department of Earth and Environmental Sciences, Università di Milano - Bicocca, Milano, Italy; 2 Department of Environmental Science and Policy, Universitá degli Studi di Milano, Milano, Italy; 3 CIMA Research Foundation, Savona, Italy; 4 Present Address: Institute for Earth Observation, Eurac Research, Bolzano, Italy; 5 Present Address: Laboratoire de Physique, ENS de Lyon, Université Claude Bernard, Université Lyon, CNRS, Lyon, France; 6 Institute of Atmospheric and Climate Sciences, CNR, Bologna, Italy

**Keywords:** Atmospheric chemistry, Atmospheric dynamics, Climate-change impacts, Hydrology, Hydrology

## Abstract

Climate change impacts are non uniformly distributed over the globe. Mountains have a peculiar response to large scale variations, documented by elevation gradients of surface temperature increase observed over many mountain ranges in the last decades. Significant changes of precipitation are expected in the changing climate and orographic effects are important in determining the amount of rainfall at a given location. It thus becomes particularly important to understand how orographic precipitation responds to global warming and to anthropogenic forcing. Here, using a large rain gauge dataset over the European Alpine region, we show that the distribution of annual precipitation among the lowlands and the mountains has varied over time, with an increase of the precipitation at the high elevations compared to the low elevations starting in the mid 20 century and peaking in the 1980s. The simultaneous increase and peak of anthropogenic aerosol load is discussed as a possible source for this interdecadal change. These results provide new insights to further our understanding and improve predictions of anthropic effects on mountain precipitations, which are fundamental for water security and management.

## Introduction

Mountains play a key role for the humanity by providing freshwater for the areas downstream, and are often referred to as “water towers” to highlight their importance in the hydrological cycle^1^. At the same time, mountain regions have peculiar dynamics that generate local effects of larger scale variations, such as global warming, producing elevation gradients of temperature and precipitation signals^2–7^. Mountains affect their own precipitation, as the orographic lifting of air masses favors condensation and cloud formation. The resulting distinctive increase of rainfall with altitude observed in many regions^8–12^ is a process typically referred to as the orographic enhancement of precipitation. This enhancement is influenced by several factors, including steepness of the terrain, slope orientation, static stability of the atmosphere and aerosols present in the air column. Some of the mentioned factors vary on geological time scales, while others depend on thermodynamical and microphysical properties, and can be influenced by climate variability and anthropogenic changes. For instance, aerosol chemical composition, size distribution, and number concentration can affect the vertical profile of air temperature^13^ as well as the nucleation and growth of cloud droplets^14^, and the recent increase of surface temperature has an elevation dependent magnitude, which can impact atmospheric stability^7^. It is thus expected that human activities can induce changes in the orographic enhancement of precipitation. Over the last decade, a few observational and modeling studies have investigated this topic. Some studies analyzed the temporal evolution of the ratio between mountain and lowland precipitation from nearby meteorological stations in Israel and USA^15,16^ and concluded that orographic precipitation decreased over the last several decades, possibly due to a rain suppression mechanism associated with anthropogenic aerosol emissions in nearby urban areas^17^. The results however are still controversial, due to the limited number of stations analized^18^. Recent regional climate model simulations^19^ indicate that summer precipitation at high elevations in the Alps increases in projections of global warming, despite the expected large scale precipitation reduction. The anomaly has been attributed to enhanced potential instability associated with amplified surface warming and increased convection at high elevations. More complex responses have been found in modeling studies accounting both for thermodynamical and dynamical effects related to climate change: variations in the storm track position play a major role in determining the orographic precipitation response^6,20^. The possibility of analyzing large observational datasets in the Alpine region is thus of great importance as it can significantly contribute to shedding light on the relationship between precipitation changes and elevation.

## Results

We report the analysis of the temporal variability of the orographic enhancement of precipitation in the Great Alpine Region (GAR, defined in the range 43°–49°N latitude and 4°–19°E longitude) over the last several decades, using a very large rain gauge dataset, with the aim of better understanding how the response of precipitation to anthropogenic forcing and climate variability depends on elevation. To this aim, we extend a methodology previously introduced^15^ by combining data from many stations into classes of homogeneous station elevation and comparing the precipitation among different classes.

**Figure 1:**
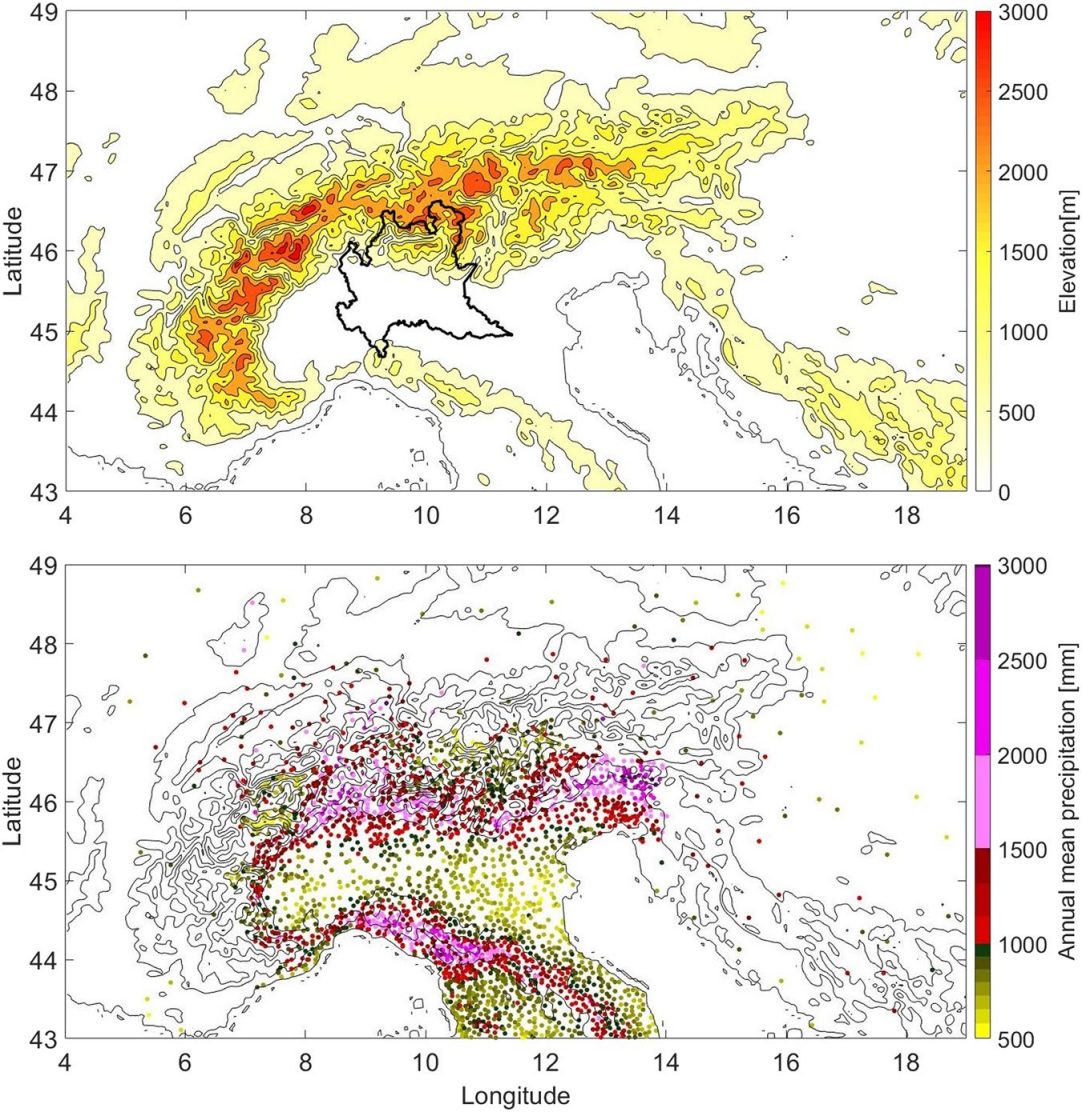
Topography (upper panel) and annual mean precipitation (1961–1990) at the different rain gauge stations (lower panel) in the Great Alpine Region. In both panels, contours are lines of constant elevation drawn every 500 m starting at sea level. The Lombardy region is shown with the black bold line in the upper panel.

The analysis has been performed over 3000 rain gauge stations in the GAR (most of them in Italy), for the period 1961–1990 (see Methods). Figure 1 shows location and mean annual precipitation at each station. The mean annual precipitation over the whole dataset is 1130 ± 14  mm (the uncertainty is the standard deviation of the GAR averaged annual precipitation over the 30 years of study) with larger values found over the mountains and lower values in the lowlands. The GAR receives storms from a wide range of directions^9^, leading to similar annual totals both on Northern and Southern Alps^21^ despite the Mediterranean influence which brings moister air on the Italian side, where precipitations are concentrated into fewer days^9^. Other regional differences exist, such as between the Western and Eastern Alps (see Supplementary information, Section S2, Figs S5 and S6), and even nearby valleys can have relatively large differences in annual precipitations^12,21^. However, in this study we want to take an approach that focuses on the main signal visible in Fig. 1, which is the elevation dependence of precipitation, rather than concentrating on the local scale variability.

**Figure 2:**
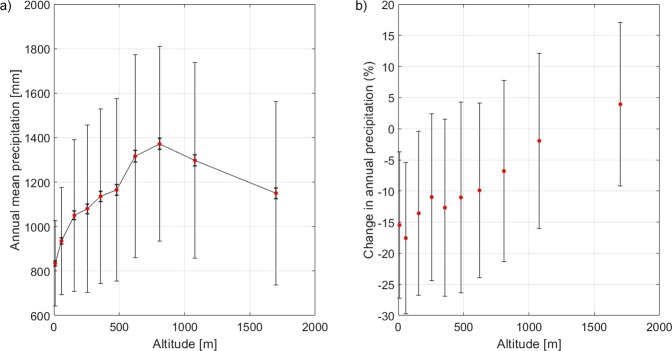
(**a**) Annual mean precipitation, relative standard deviation (fine error bars) and relative standard error of the mean (bold error bars) for each class of altitude. The full line is the linear interpolation of the points, and it represents the model used to normalize precipitation data for the Lombardy region, according to each station elevation (as described in the Methods). (**b**) Percentage variation in annual precipitation in the studied 30 year period (1961–1990), for different elevation classes. These variations have been computed from linear trends over the 30 year period. Error bars represent one standard deviation of the percentage variation in annual precipitation calculated on each station.

To this end, we consider all stations at disposal in the study area, regardless of the steepness of the terrain, the slope orientation, and their location, and cluster data depending on station elevation (as described in the Methods section). Averages are then computed within each class of altitude: a robust relationship between annual mean precipitation and elevation is evident, as shown in Fig. 2a, and it holds also for each individual season (Supplementary Material, Section S1). Maximum annual precipitation is found at elevations around 800 m, indicating that, as expected, precipitation is not a monotonic function of elevation and other factors, both local and regional, are important in setting the rainfall. Mean annual precipitation over 1540 stations at altitudes above 400 m (in the following referred to as mountain stations) is 1256 mm, while in the 1462 lowland stations (below 400 m of elevation) it is 1005 mm. Thus, the time averaged orographic enhancement of precipitation, defined as the ratio between mountain precipitation to lowland precipitation, is $\overline{\,\mathrm{Ro}\,}=1.25$ in this dataset. The goal of the subsequent analysis is to determine whether the orographic enhancement of precipitation is stable over the years or whether it changes over time.

**Figure 3:**
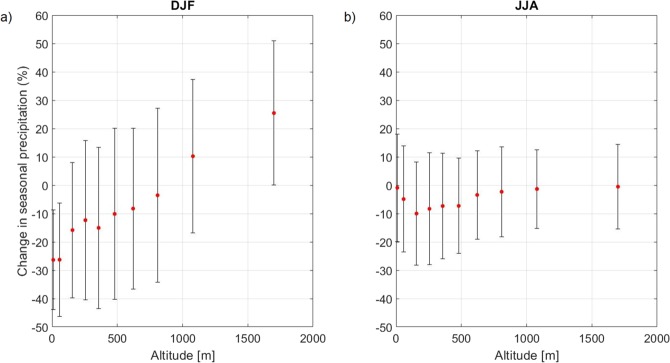
Relative change in seasonal precipitations as function of station elevation, for the period 1961–1990: (**a**) winter (December, January, February); (**b**) summer (June, July, August).

The analysis of the 30 year long precipitation time series reveals that there is a weakly negative linear trend of annual precipitation between 1961 and 1990, which, considering the large interannual variability of precipitation in the GAR, is not statistically significant. However, a clear dependence of this tendency on station elevation is found (Fig. 2b): while the linear trend accounts for a reduction of annual precipitation at low elevations of about 15% over the 30 year period, at high elevations there is no change. The elevation dependence of the fractional change of precipitation over the 30 year long period is robust and significant at the 99% confidence level. A seasonal analysis reveals that such elevation gradient is particularly strong during winter, when sea level stations present a 25% reduction and the highest stations a 25% increase of precipitation, and absent during summer (Fig. 3), with intermediate behavior for spring and fall (Supplementary Information, Section S1 and Fig. S2). The elevation dependent change is evident in different GAR subregions (Supplementary Information, Section S2 and Fig. S7).

**Figure 4:**
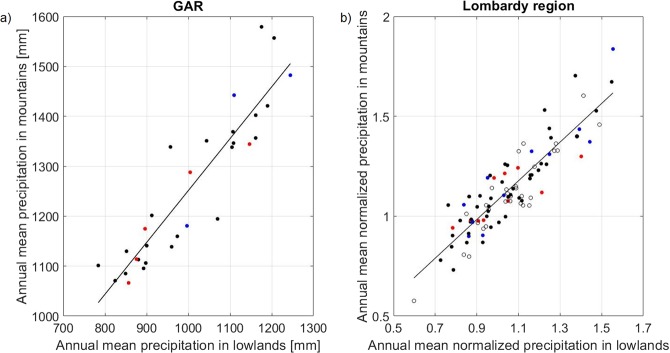
(**a**) Scatter plot of the yearly precipitation averaged over the mountain and lowland stations in the GAR. (**b**) Scatter plot of the yearly normalized precipitation averaged over the mountain and lowland stations in the Lombardy region. Each point corresponds to the values of a given year (in the 1961–1990 period for panel a, and in the 1920–2017 period for panel b). Solid lines show the linear interpolation of the data. In both cases the correlation coefficients between the mountain and lowland annual precipitation time series (equal to 0.9 in both panels) are statistically significant at the 99% confidence level. The colours of the points represent the phase of the NAO of the year to which they refer: positive phase (red), negative phase (blue) and neutral phase (black). The circles represent points whose NAO phase is not available.

The tendencies in seasonal and annual precipitation discussed above emerge out of a signal that has large interannual variability: variations in the large-scale atmospheric circulation, in part associated with climatic modes of variability such as the North Atlantic Oscillation (NAO) and the Atlantic Multidecadal Oscillation (AMO), impact weather patterns and cause year-to-year fluctuations of annual precipitation in the region, which has a standard deviation of about 20% of its mean over the 30 year long period investigated. A latitudinal shift of the storm track results in large precipitation differences between NAO positive and negative phases, with enhanced precipitation over the GAR region during the NAO- phase, especially during the winter season^22,23^ (see also Supplementary Information, Section S3). The influence of AMO is less clear: summer precipitation has been shown to be larger during the warm phase of the AMO^24^ while spring precipitation anticorrelates with AMO^23^, probably due to changes in weather type frequency^25^. In our data series, however, no link between precipitations and AMO has been found (Supplementary Table S5), probably due to the short length of the time series compared to the long period of the AMO. The weather pattern changes associated with climate modes of variability induce large-scale dynamical and thermodynamical effects which generate a coherent precipitation signal over the GAR, as indicated by the significative and large correlation between year-to-year fluctuations of precipitation in different subregions of the GAR (see Table S3). Consistently, the interannual variability is similar between lowland and mountain stations, as shown by the scatter plot of mean annual precipitation above and below 400 m of altitude (Fig. 4a), which have a correlation coefficient of 0.9. While annual precipitation time series for mountains and lowlands have both a standard deviation of about 20% of their respective mean (0.21 and 0.18, respectively), the ratio between them, *Ro*, is much more stable over time, with a standard deviation of about 5% of its mean value, and is not significantly correlated with neither NAO nor AMO (see Supplementary Information Section S3 and Tables S4, S5). Investigating this ratio, thus, increases the signal-to-noise ratio and provides a tool for detecting long term changes in the orographic enhancement of precipitation, if present.

**Figure 5:**
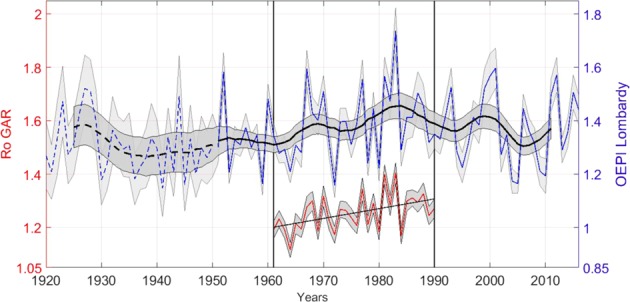
Time series of the orographic enhancement of precipitation over the whole GAR (Ro) for the period 1961 to 1990 and over the Lombardy region (OEPI). In the time series of the OEPI over the Lombardy region, dashed lines refer to the period before 1951 in which the low number of stations available makes the series significantly less reliable. The grey band on the time series represents the standard error of the mean for each annual value. A 11-year window, 3-year standard deviation Gaussian filter (black line) is shown with its twice standard deviation on the filter (95% confidence limits), calculated through the propagation of uncertainty. The uncertainty used is the standard error of the mean (grey band on the filter). The trend over the period 1961–1990 (indicated by the thin black line in the lower portion of the figure) is significant at the 99% confidence level and its value is 0.004 yr^−1^, leading to an increase of the orographic enhancement of precipitation from 1.20 to 1.31 over the 30 year period.

As shown in Fig. 5, a positive trend in *Ro*, which is statistically significant at 99% confidence level, is found over the period 1961–1990 (0.004 yr^−1^), indicating that the orographic enhancement of precipitation in the Alpine region has increased from 1.20 ± 0.01 to 1.31 ± 0.01 in 30 years. In other words, the annual precipitation averaged over mountain stations is 20% larger than in lowland stations at the beginning and it becomes 31% larger than in lowland stations at the end of the 30 year study period. The variation is mainly related to winter precipitation, while no elevation dependent signal is observed during summers (see Fig. S3 in the Supplementary material). This statistically significant change needs to be put into a longer term context, in order to verify whether it is part of a long time trend or not.

With the aim of extending the above analysis to several decades long time series, we use all available stations in the Lombardy region (Central Alps, Italy), regardless of their time of operation. Since the available stations change with time, a procedure to reduce biases introduced by changing the location of the operating stations at which data are collected is necessary: to this aim, annual precipitation at any given station is normalized by the long term mean precipitation at its elevation (see Methods). The average annual mean normalized precipitation has been computed for all mountain and lowland stations, separately (see Fig. 4b).

The Orographic Enhancement of Precipitation Index (OEPI) is introduced, defined as the ratio of the normalized annual precipitation averaged over mountain stations to the normalized annual precipitation averaged over lowland stations, and then multiplied by the 30 year long ratio between mountainous precipitation and lowland precipitation previously found (1.25). The OEPI time series from the Lombardy region stations is shown in Fig. 5. The orographic enhancement of precipitation shows consistent behaviour over the two datasets in the overlapping period, increasing for about three decades from 1950s to 1980s and then it decreases until today. In other words, in the 1980s the difference in annual precipitation between high elevation stations and low elevation stations has reached the largest values over the last 70 years. We do not make any statement about the value of the index before 1950 as the number of available stations is very low prior to this date (Supplementary Fig. S8).

## Discussion

A straightforward definition of the main factors driving the observed change is not possible at present, as many mechanisms could be responsible for this behaviour. Clearly, modifications in the airflow direction and intensity during storms might modify the spatial distribution of precipitation in the region and thus the relationship between precipitation and elevation. Decadal changes in weather type occurrence have been documented^26–28^ and cannot be ruled out as the origin of the observed changes in the orographic amplification of precipitation. However, it is interesting to note that the elevation dependent precipitation change is found both on the Northern and Southern Alpine slopes, as well in Western, Central, and Eastern Alps separately, albeit with more scattered results likely associated to the reduced statistics when subregions are analyzed (Supplementary Information, Section S2 and Fig. S6). This suggests that the mechanism responsible for this elevation dependent precipitation change could be independent of dynamical variations. In this regard, we note that the peak in the orographic enhancement of precipitation occurs at the same time as the maximum air pollution in the region: starting in the 1980s, new regulations led to the decline in anthropogenic aerosol concentration in the GAR, which caused a significant increase of the solar radiation reaching the lowlands^29–31^. This brightening has been considered to be at the basis of the peculiar elevation dependent warming observed over the last decades in the Alpine Region: while in the Himalayas and in the Rockies warming has been faster at higher elevations, in the highly urbanized Alpine region warming has been faster in the lowlands, likely because of the increased shortwave radiation reaching the surface as air pollution decreased^32^. The possibility of a link between elevation dependent precipitation changes and aerosol loads is corroborated by the fact that the signal is strongest during the winter season, when the boundary layer is shallowest and the pollution reaches high levels near the ground^33^, and absent during summer, when the boundary layer depth reaches 1000 m and air becomes cleaner (see both Fig. 3 and Supplementary Information S1). A varying aerosol concentration has potential impacts on the orographic enhancement of precipitation essentially for two effects: the corresponding change in atmospheric stability and the possible change in the microphysical properties of clouds. Along the first line, radiative effects of aerosols can induce a surface cooling and an accompanied warming at the levels in which solar radiation is absorbed by opaque aerosols such as black carbon. The net effect on air column stability is however not simply determined and it should be assessed performing modeling studies. Along the second line, the indirect effect of aerosols on precipitation is still unclear: the change of the number concentration, chemical properties, and size distribution of aerosols affect cloud microphysics by influencing the properties of cloud condensation nuclei and of ice nuclei. While there is a general agreement that high aerosol loads lead to a reduced efficiency of collision/coalescence of droplets suppressing precipitations in warm clouds^14,34^, the situation is much more elaborate in mixed-phase clouds, where aerosols acting as ice nuclei might on the contrary increase the precipitation efficiency^35–37^. At present it is thus unclear what process would dominate in determining the response of precipitation to varying aerosol loads. Our results seem to indicate that the increased pollution might have increased the orographic enhancement of precipitation, but it is not clear whether this should be related to a suppression of precipitation in the heavily polluted lowlands or an increased precipitation at higher elevations.

This study demonstrates that the orographic enhancement of precipitation dependence on anthropogenic forcing is an important research line, and more observational data and modeling work are necessary in order to quantify the effects of both aerosols and global warming on it, which will allow us to make more reliable predictions of precipitation and water storage in the mountain regions in the coming decades. It will also be important to assess whether the distribution of precipitations and extreme conditions (such as heavy rains, consecutive wet days, consecutive dry days) present long term variability that systematically depends on elevation.

## Methods

In this work two different precipitation datasets have been used. The first one is a high density monthly dataset over Italy and the Alpine region for the period 1961–1990^12^. This dataset is the result of more than 10 years of activities carried out at the Institute of Atmospheric Sciences and Climate of the Italian National Research Council (ISAC-CNR) and at the Department of Environmental Science and Policy at Milan University to obtain the largest possible amount of precipitation records and metadata for Italy and the surrounding areas. To this aim, many different datasets have been included. Starting in early 1990s, many mechanical rain gauges have been dismissed and replaced with automatic instruments. In most cases, this has been done without maintaining the old and the new instruments operative at the same time, to calibrate the new instruments, and for this reasons the time series cannot be merged to generate longer time series. Moreover, precipitation data have been collected at the national level for several decades and the operations have been regionalized at the end of the 20^*th*^ century. For these reasons, the period 1961–1990 has been chosen to guarantee a degree of homogeneity of data availability. Careful quality checks and data filling have been performed^12^. At the end of this procedure, the dataset provides monthly precipitation at every station for the 30 year long period with no gaps. The number of available stations in the area considered in this paper is 3002.

A second dataset is comprised of historical time series of monthly precipitation for 561 stations in the Lombardy region. This dataset has been created at the Department of Environmental Science and Policy of University of Milan by analyzing and digitizing a large amount of historical data archives (monographic studies, bulletins, reports, etc.) and by adding data of local meteorological networks of ARPA (Agenzia Regionale per la Protezione Ambientale) Lombardia and CMG (Centro Monitoraggio Geologico). In a second step available time series have been homogenized to remove non-climatic changes in the data: in the last decade, the scientific community has become aware of the fact that the real climate signal in original series of meteorological data is generally hidden behind non-climatic noise caused by station relocation, changes in instruments and instrument screens, changes in observation times, observers, and observing regulations, algorithms for the calculation of means and so on. So, at present, the statement that time series of meteorological data cannot be used for climate research without a clear knowledge about the state of the data in terms of homogeneity has a very large consent^38^.

Different stations have data available for different time periods and some data between the starting and end times of each station are missing. So a preliminary treatment of the dataset has been necessary. For each station, a climatological monthly precipitation was computed. We then considered, for each station, only years in which at least 9 months of data are present and we replaced the missing ones by the corresponding climatological value. We then computed the annual and the seasonal precipitation at each station by summing over the considered months. At the end of the procedure we obtained a dataset of precipitation with a variable number of stations every year. In the Supplementary Fig. S8 the number of available stations at elevations above and below 400 m for every year is shown: the number of available stations changes over time and it has a big rise from about 20 to about 100 in 1951, when historical data archives have been started to be digitized. In this work, the focus has been on the period from 1951 onward in order to have a relatively stable statistics. Each of those time series has been normalized, either on its own mean value of annual precipitation and on an estimate of the mean annual precipitation appropriate for its elevation, as described below.

A model of the elevation dependent annual mean precipitation has been constructed: using the high density database for the 1961–1990 period, we have divided the data in 10 classes based on station altitude, where the class limits are chosen so that each class has the same number of stations (300). The annual mean precipitation for each station has been computed and then the mean value of the annual mean precipitation over the 300 stations in the same altitude class has been computed, together with its standard deviation, and its standard error. The results of this procedure are shown in Fig. 2a. A linear interpolation between nearby classes has been performed to define the annual mean precipitation at each elevation between the central value of each class.

This model has then been used to normalize the precipitation data from the Lombardy region. To this aim, the model annual mean precipitation corresponding to the elevation of each Lombardy station has been calculated. The annual precipitation of each station in the Lombardy region has then been divided by these model values. We refer to this dataset as the Lombardy region normalized dataset. To verify that results are not sensitive to the details of the normalization procedure, we also used as normalizing factor the mean precipitation of the individual Lombardy station. The results are similar and all the figures presented in the paper refer to the former normalization method.

Lombardy stations have then been clustered in two classes, based on whether their elevation is above or below 400 m. This allows to form two classes with nearly equal number of stations (see Supplementary Fig. S8). We varied the precise value of the elevation threshold used to define mountain and lowland stations to verify that the results do not show a strong sensitivity. A time series has been constructed for each of the two classes, taking for each year the average of the normalized annual precipitation over all the available station in each class. The two obtained (adimensional) time series have then been multiplied by the mean annual precipitation above and below 400 m derived from the 1961–1990 homogeneous time series (1256 mm and 1005 mm, respectively). The ratio between the resulting dimensional time series is the Orographic Enhancement of Precipitation Index, as shown in Fig. 5.

The NAO monthly index time series (from 1950 to 2018) has been retrieved from the NOAA website (https://www.cpc.ncep.noaa.gov/products/precip/CWlink/pna/nao.shtml) and the AMO monthly index time series (from 1948 to 2019) from NOAA PSD website (https://www.esrl.noaa.gov/psd/data/timeseries/AMO). Annual and seasonal indices have been obtained by averaging the monthly index over the appropriate months. Positive and negative NAO phases have been defined as values of the index whose distance from the mean is larger than the standard deviation.

To best fit the dataset, the least-square method is used and the significance of the linear trends is estimated using the one-tail Student’s t test at the 95% confidence level^39^. The correlation between (detrended) variables has been calculated through the Pearson correlation coefficient which measures their linear dependence. The significance of the correlations with the climatic modes of variability has been estimated by verifying whether the null hypothesis of lack of correlation can be rejected at the 95% confidence level, using the Student’s t test^39^.

## Supplementary information

Supplementary Information

## Data Availability

The datasets generated and analysed during the current study are available from the authors on reasonable request.
