# Supplementary material for: Variability of orographic enhancement of precipitation in the Alpine region
Source: Sci Rep. 2019 Sep 16;9:13352. doi: 10.1038/s41598-019-49974-5 (PMC6746858; doi:10.1038/s41598-019-49974-5)
Supplement: Supplementary file 1 — Supplementary Information [file 41598_2019_49974_MOESM1_ESM.pdf]

Supplementary Material for Variability of orographic enhancement of precipitation in the Alpine region

Anna Napoli[1,2,3], Alice Crespi[2,4], Francesco Ragone[1,5], Maurizio Maugeri[2,6], and Claudia Pasquero[1,6]

[1]Department of Earth and Environmental Sciences, University of Milano - Bicocca, Italy
[2]Department of Environmental Science and Policy, Universitá degli Studi di Milano, Italy
[3]CIMA Research Foundation, Savona, Italy
[4]Institute for Earth Observation, Eurac Research, Bolzano, Italy
[5]Laboratoire de Physique, ENS de Lyon, Universit Claude Bernard, Universit Lyon, CNRS, Lyon, France
[6]Institute of Atmospheric and Climate Sciences, CNR, Italy

S1 Seasonal precipitations

Precipitations in the Great Alpine Region (GAR) have a robust seasonal cycle, with a deep minimum in winter and two peaks (late Spring and Fall). Summer precipitations are often associated with thunderstorms and regulated by convective processes, while precipitations in the rest of the year are associated with typical midlatitude storms, linked to synoptic scale perturbations[3]. The relation between seasonal precipitation and elevation, computed following the same procedure as for the annual precipitation presented in the main paper, is shown in fig. S1 for the different seasons: Spring (March, April, May, MMA), Summer (June, July, August, JJA), Autumn (September, October, November, SON), and Winter (December, January, February, DJF). It can be seen that the precipitation in summer is a monotonic function of elevation, while in all other seasons it reaches a maximum at about 800 m and then it decreases. This is probably due to the the different nature of the precipitations.
Similarly, the relative change of precipitation calculated over the 1961-1990 period has seasonal differences, shown in fig. 3 of the main paper for winter and summer, and here for spring and fall (fig. S2). The mean change of precipitation over the GAR has been very different in the different seasons (a strong

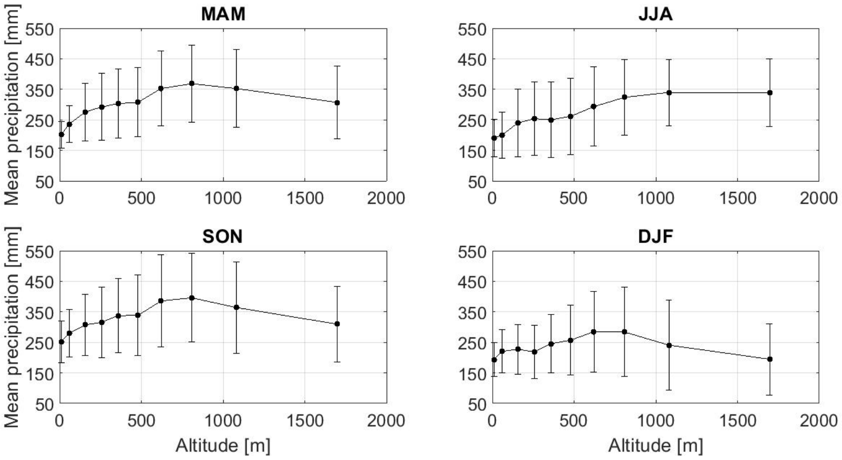

Figure S1: Seasonal mean precipitation and relative standard deviation for each class of altitude; each panel refers to a different season.

decrease of fall precipitation, an increase of spring precipitation, and weak reduction for summer and winter precipitations), but, rather than focusing on the mean variations, we are interested here in the dependence of the changes on elevation. The largest dependence is present in winter, no elevation dependence exists in summer, and intermediate behaviors are found in spring and fall. We thus perform a separate analysis for summers, and cluster together the other seasons that have a similar dependence on elevation, defining an extended winter season from September to May (SONDJFMAM) of the following year. The use of the extended winter season allows us to use a larger dataset than for winter only, thus obtaining results that are statistically more significant. We report in fig. S3 the analogue to fig. 5 of the main paper, for extended winter and for summer separately. Over the period 1961-1990 there is no statistically significant trend in the value of Ro, the ratio of mountain to lowland precipitations, during summers (trend = 0.0016 yr^{-1}, p-value=0.65), while the trend is significant and positive (trend=0.004 yr^{-1}, p-value=0.002) during extended winters. For DJF only, the trend is considerably stronger (0.013 yr^{-1}) but the large year-to-year variability reduces the confidence of the result (p-value=0.40, figure not shown).

S2 Partition of the Great Alpine Region in subregions

With the aim of analyzing whether there are important differences in the precipitation over different geographical areas, we partition the stations inside the box 43°-49° N and 4°-16° E in different groups (see fig. S4). First of all, we

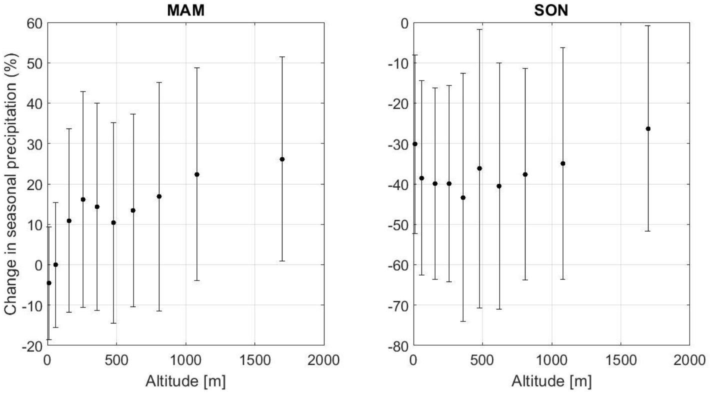

Figure S2: Relative change of seasonal precipitation in the GAR over the 1961-1990 period as a function of elevation, for (left panel) spring and (right panel) fall.

divide the Alps from the Apennines, and then each of the two regions is further partitioned according to the relative watershed. We thus have the Northern and the Southern flanks of the Alps, and the Northeastern and Southwestern flanks of the Apennines. The Alps are also divided in three subregions: Western, Central, and Eastern Alps.
Latitude and longitude coordinates of fig. S4 are shown in Table S1, the relative number of stations in Table S2.

	Western Alps	Central Alps	Eastern Alps	Apennines
Min latitude	43°N	45°N	45°N-43°N	43°N
Max latitude	49°N	49°N	49°N	45°N
Min longitude	4°E	8°33'E	11°24'E-14°E	8°33'E
Max longitude	8°33'E	11°24'E	16°E	14°E

Table S1: Border latitude and longitude of the different subregions of fig. S4.

Regions	n. stations
Western Alps	532
Central Alps	735
Eastern Alps	530
Apennines	1183
Northern Alps	408
Southern Alps	1389
Northeastern Apennines	607
Southwestern Apennines	576

Table S2: Number of stations for each subregion.

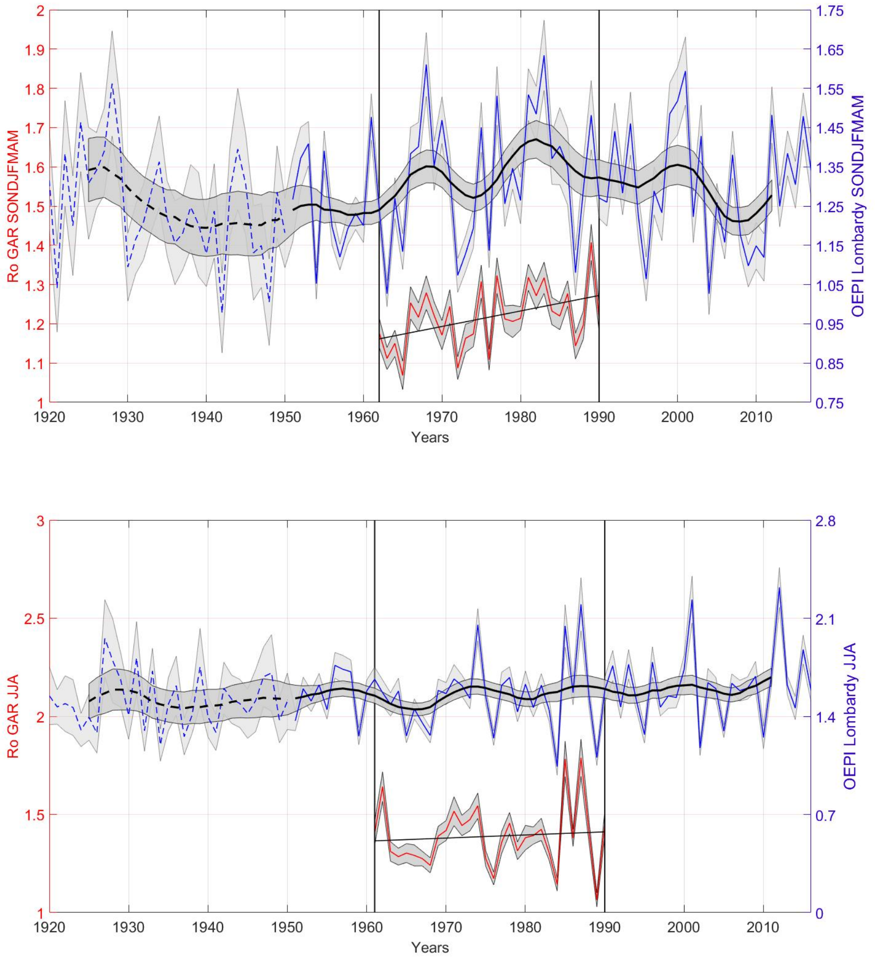

Figure S3: Time series of the orographic enhancement of precipitation over the whole GAR (*Ro*) for the period 1961 to 1990 and over the Lombardy region (OEPI), analogous to fig. 5 in the main paper but performed for different seasons (extended winter, top panel; summer, bottom panel).

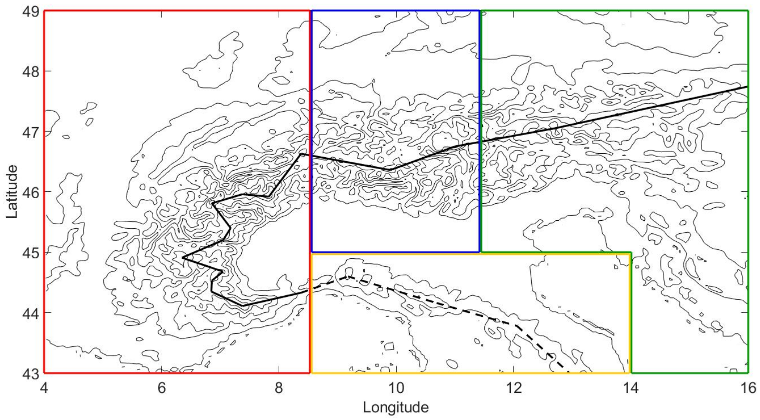

Figure S4: Different subregions used in the study: Western Alps (red box), Central Alps (blue box), Eastern Alps (green box), Apennines (yellow box). The thick black lines indicate the Alpine (full line) and Apennines (dashed line) watersheds. Thin black contour lines are lines of constant elevation, drawn from sea level every 500 m.

Within each subregion, the same analysis presented in the main paper is performed. The stations are clustered depending on their elevation, and the mean annual precipitation is computed for each elevation class. The corresponding standard deviation derives from the scatter of the climatological annual mean precipitation at each station in the same class. Results are presented in fig. S5. There clearly are differences among the different regions. For instance, precipitations on the Southward side of the Alps are larger than precipitations on the Northward side, and the same difference is found between the Southwestern and the Northeastern side of the Apennines. This is related to the larger moisture content of the air masses arriving on the ridges from the South, which have traveled over the Mediterranean Sea, that lead to enhanced precipitation on the windward side of the mountain range. Also, a precipitation gradient exists from West to East, with larger precipitations in the Eastern Alps, and the Southwestern Apennines have a relatively constant mean precipitation over the lowest 400 m from sea level. Despite those differences, many common features are clearly visible. The increase of precipitations with elevation is present in all subregions, up to an elevation of 600 to 1000 m, and then a decrease occurs at higher elevation. In the Apennines, the reduction is not present, probably due to the much lower elevation of the peaks in this mountain range compared to the Alpine range.

Time series of annual precipitation in the different subregions are all significantly correlated among each others and with the mean GAR time series (see table S3), indicating that the interannual variability of precipitation is, as expected, largely associated to large scale atmospheric dynamics rather than to

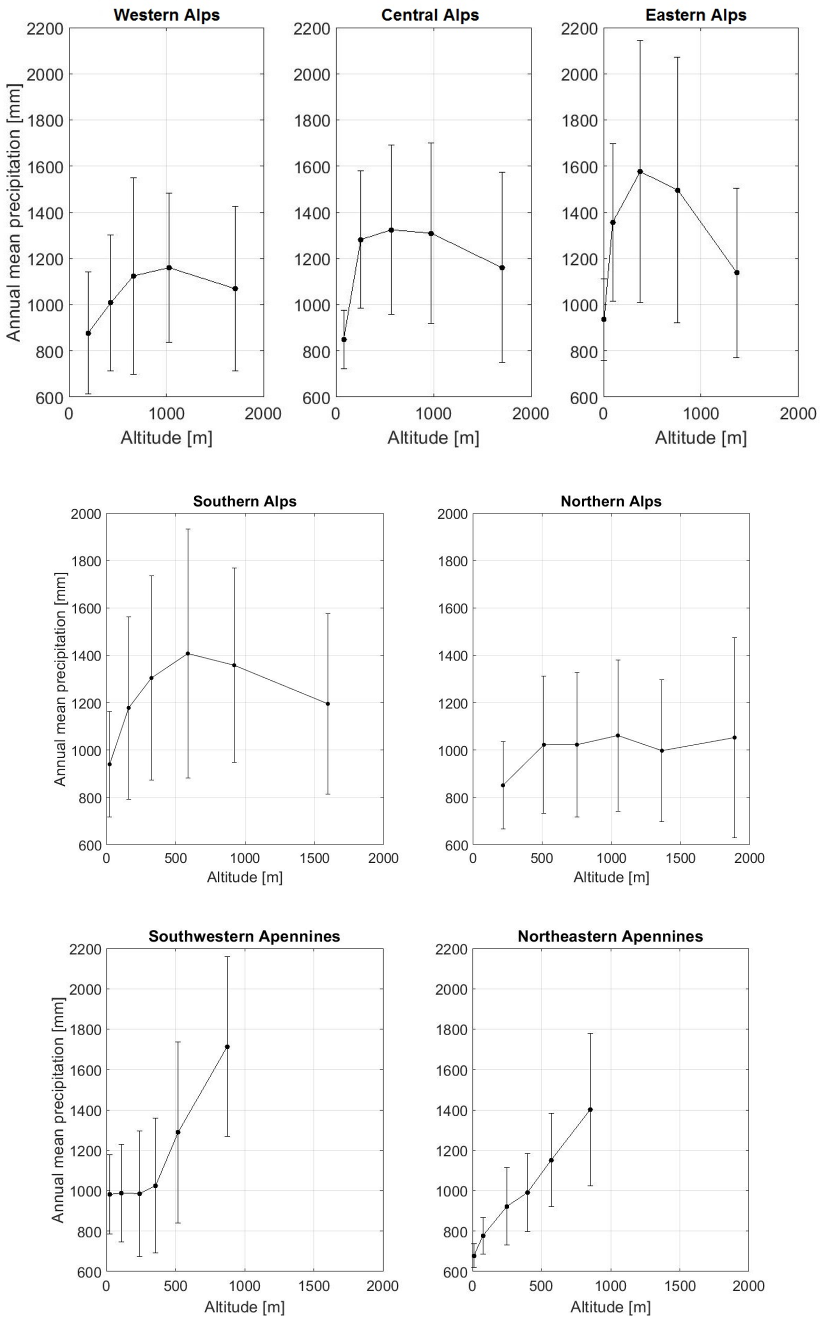

Figure S5: Annual precipitation as function of elevation in the three Alpine subregions (top panels), the two Alpine watersheds (middle panels) and the two Apennines watersheds (bottom panels). Points indicate the mean value and errorbars indicate the standard deviation computed over the different stations in each class. The total number of station in each subregion is indicated in Table S2.

more local effects. Linear trends computed over the 30 year long study period are shown in fig. S6. All regions except the Apennines show a similar elevation dependent precipitation change, with lowlands having a reduction of precipitations, and higher elevations having a weaker decrease or even an increase of annual mean precipitations. The Apennines, on both sides of the watershed, experience a reduction of precipitation without a clear dependence on elevation. During winter, the season during which the elevation dependent precipitation change is more evident, a weak dependence on elevation is found, even in the Apennines (fig. S7). Note that the use of subregions forces us to reduce the number of observations in each geographical class. As a consequence, results obtained in each subregion are noiser than when the GAR is considered as a whole. Note also that few stations are available for this study on the Northern flank of the Alps. It is thus expected that the signal for this region is less robust (in a statistical sense).

**Region1**	**Region2**	**Corr. coeff.**
Western Alps	Central Alps	**0.77**
Western Alps	Eastern Alps	**0.42**
Central Alps	Eastern Alps	**0.70**
Apennines	Eastern Alps	**0.77**
Apennines	Western Alps	**0.55**
Apennines	Central Alps	**0.65**
GAR	Western Alps	**0.77**
GAR	Eastern Alps	**0.83**
GAR	Central Alps	**0.89**
GAR	Apennines	**0.90**

Table S3: Correlation between time series of annual precipitation in the different regions among each others and with the mean GAR time series. All the correlations are significant at least at the 95% confidence level.

S3 The link with the North Atlantic Oscillation

It is well known that annual mean precipitations in Europe are modulated by the North Atlantic Oscillation (NAO). Negative phases of the NAO are associated with a southward shift of the storm track, which then brings storms and perturbations to Southern Europe, leading to higher precipitations over the GAR region [1, 2]. The link between precipitations and NAO is particularly present during winters and absent during summers [4]. For this work, the NAO monthly index time series has been retrieved from the NOAA website[1], and annual and seasonal indices have been obtained by averaging the monthly values over the period of interest. Annual precipitations over the GAR region in

[1]https://www.cpc.ncep.noaa.gov/products/precip/CWlink/pna/nao.shtml

the period 1961-1990 are not significantly correlated with NAO (see table S4). Extended winter precipitations are correlated with NAO at the 98% confidence level, and the correlation coefficients are similar for lowlands and for mountains (-0.49 and -0.44, respectively), indicating that precipitations are enhanced during the negative NAO phase at all elevations. The time series of the mountain to lowland precipitation ratio is not significantly correlated with NAO. We conclude that the large interannual variability of precipitation associated with the NAO is effectively removed from the orographic enhancement of precipitation, Ro, as defined in the paper. Similar conclusions can be drawn analyzing DJF only (winter rather than extended winter season) as well as different subregions separately, but with lower statistical significance probably due to the limited dataset obtained when the data are subsampled. Summer precipitations are not significantly correlated with NAO, but the ratio between mountain to lowland summer precipitation has a negative correlation with NAO. Despite this is not at the base of the increase of Ro during the 1961-1990 period (which is absent during summers), we think it might be interesting to further investigate this aspect in the future. Summer precipitation during NAO+ phases is almost entirely of convective origin, while during NAO- phase it is also of synoptic scale origin, and the two precipitation types can have a different elevation dependence. The use of numerical simulations can be particularly useful as it allows the separation of convective from synoptic scale precipitation.

References

[1] Hurrell, J. W. (1995). Decadal trends in the north atlantic oscillation: regional temperatures and precipitation. *Science*, 269(5224):676–679.

[2] Hurrell, J. W. and Deser, C. (2010). North atlantic climate variability: the role of the north atlantic oscillation. *Journal of Marine Systems*, 79(3-4):231–244.

[3] Pieri, A., von Hardenberg, J., Parodi, A., and Provenzale, A. (2015). Sensitivity of precipitation statistics to resolution, microphysics, and convective parameterization: A case study with the high-resolution wrf climate model over europe. *Journal of Hydrometeorology*, 16(4):1857–1872.

[4] Qian, B., Corte-Real, J., and Xu, H. (2000). Is the north atlantic oscillation the most important atmospheric pattern for precipitation in europe? *Journal of Geophysical Research: Atmospheres*, 105(D9):11901–11910.

Region	Season	Value	Corr. coeff.
GAR	Annual	mountain	-0.32
		lowland	-0.17
		Ro	-0.31
	DJF	mountain	**-0.48**
		lowland	**-0.51**
		Ro	+0.17
	SONDJFMAM	mountain	**-0.49**
		lowland	**-0.44**
		Ro	-0.32
	JJA	mountain	-0.32
		lowland	-0.11
		Ro	**-0.37**
Lombardy	Annual	Ro	+0.03
	DJF	Ro	+0.18
	SONDJFMAM	Ro	+0.07
	JJA	Ro	**-0.38**

Table S4: Correlation with NAO of seasonal and annual precipitations over the GAR and Lombardy region for mountains, lowlands and Ro. The correlation is calculated between detrended variables. Correlation coefficients significant at the 95% confidence level (as discussed in the section "Methods" of the main paper) are shown in bold. Correlations with GAR have been computed over the 1961-1990 period. Correlations with Lombardy region have been computed over the 1950-2016 period.

Region	Season	Value	Corr. coeff.
GAR	Annual	mountain	-0.1
		lowland	-0.12
		Ro	+0.07
	DJF	mountain	-0.1
		lowland	-0.22
		Ro	+0.29
	SONDJFMAM	mountain	+0.01
		lowland	+0.02
		Ro	+0.04
	JJA	mountain	+0.09
		lowland	+0.05
		Ro	+0.06
Lombardy	Annual	Ro	-0.08
	DJF	Ro	-0.18
	SONDJFMAM	Ro	-0.16
	JJA	Ro	-0.004

Table S5: Correlation with AMO of seasonal precipitations over the GAR for mountains, lowland and Ro and over the Lombardy region for Ro. The correlation is calculated between detrended variables. None of the correlations is statistically significant. Correlations with GAR have been computed over the 1961-1990 period. Correlations with Lombardy region have been computed over the 1950-2016 period.

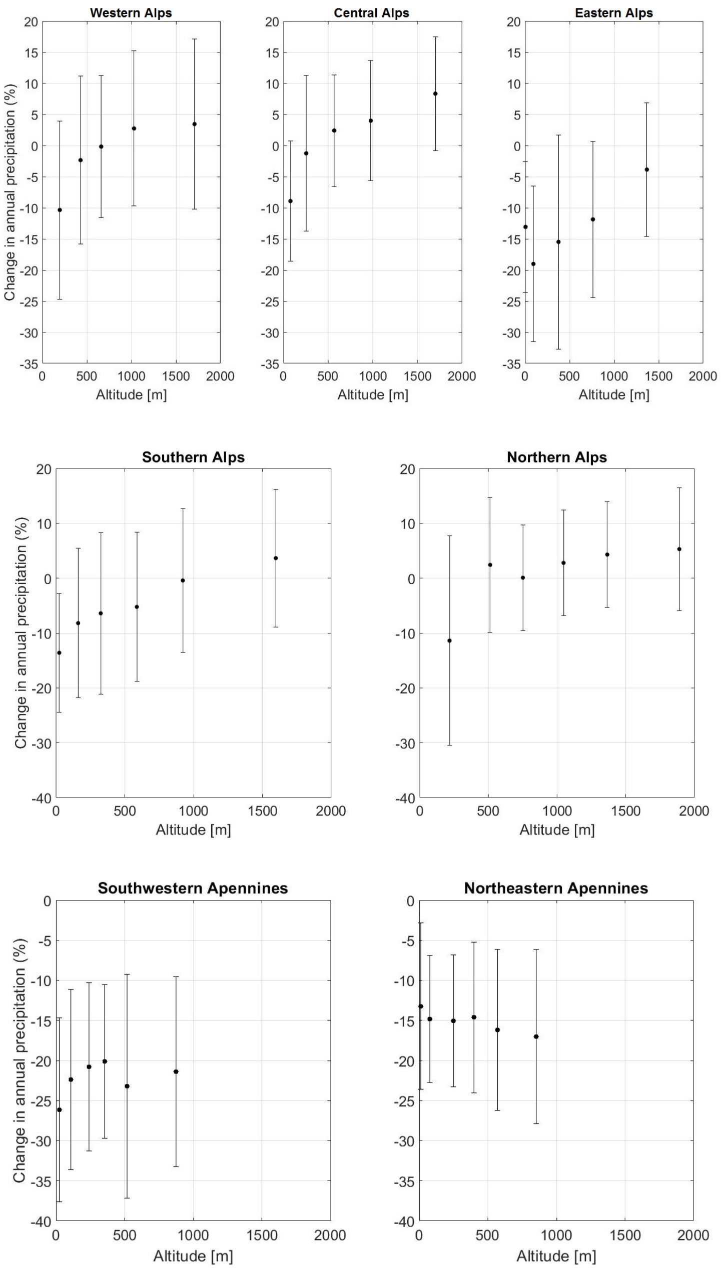

Figure S6: Relative change of annual mean precipitation for the studied 30 year period (1961-1990), for different elevation classes. These variations have been computed from linear trends over the 30 year period. Error bars represent the standard deviation of the percentage variation in seasonal precipitation calculated on each station. Top panel: Alps, partitioned into Western, Central, and Eastern Alps. Middle panel: Alps, partitioned according to the watershed. Bottom panel: Apennines, partitioned according to the watershed.

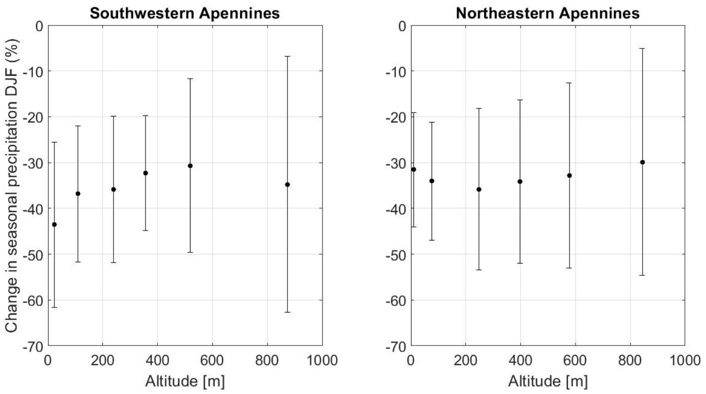

Figure S7: Same as fig. S6, bottom panels (the two watersheds of the Apennines), but for winter (DJF) only.

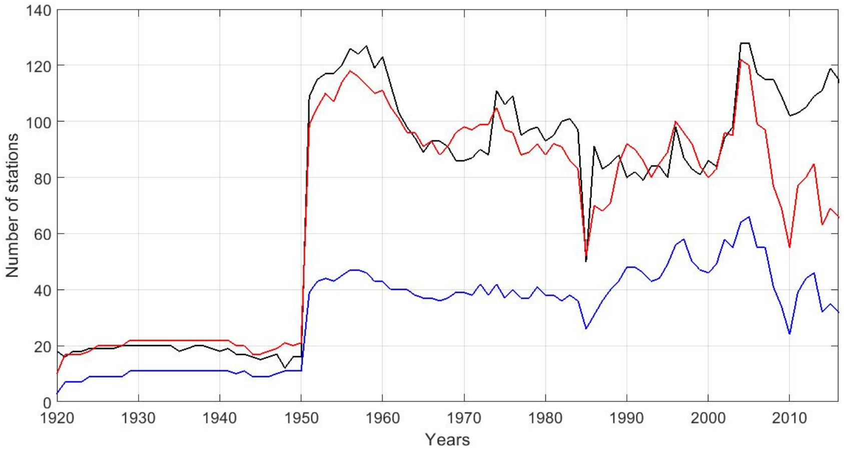

Figure S8: Number of available stations within the Lombardy region for every year in each class of altitude: height more than 1000 m (blue line), more than 400 m (red line) and less than 400 m (black line).
